# Quantitative reconstruction of neuronal mitochondrial network in neurites and somata in rat hippocampus and prefrontal cortex

**DOI:** 10.3389/fphys.2026.1735677

**Published:** 2026-04-21

**Authors:** Lu Wang, Linlin Li, Jiazheng Liu, Zichen Wang, Jing Liu, Sheng Chang, Jingbin Yuan, Xi Chen, Qiwei Xie, Lijun Shen, Xianhua Wang, Gang Li, Heping Cheng, Hua Han

**Affiliations:** 1 Institute of High Energy Physics, Chinese Academy of Sciences, Beijing, China; 2 National Biomedical Imaging Center, State Key Laboratory of Membrane Biology, Institute of Molecular Medicine, College of Future Technology, Peking University, Beijing, China; 3 Key Laboratory of Brain Cognition and Brain-inspired Intelligence Technology, Institute of Automation, Chinese Academy of Sciences, Beijing, China; 4 Team of Microscale Reconstruction and Intelligent Analysis, Laboratory of Brain-AI, Institute of Automation, Chinese Academy of Sciences, Beijing, China; 5 School of Future Technology, School of Artificial Intelligence, University of Chinese Academy of Sciences, Beijing, China; 6 Research Unit of Mitochondria in Brain Diseases, Chinese Academy of Medical Sciences, PKU-Nanjing Institute of Translational Medicine, Nanjing, China; 7 Academy for Advanced Interdisciplinary Studies, Peking University, Beijing, China

**Keywords:** 3D volumetric reconstruction, hippocampus, mitochondria, nanotunnels, the prefrontal cortex

## Abstract

**Introduction:**

Mitochondrial networks exhibit striking heterogeneity in their morphology and distribution across different neuronal compartments, reflecting the diverse metabolic demands of these structures.

**Methods:**

In this study, we used automated tape-collecting ultramicrotome scanning electron microscopy (ATUM-SEM) to reconstruct and quantify mitochondrial networks in the somata and neurites of neurons in the rat prefrontal cortex (PFC) and hippocampus (HPC; CA1 stratum radiatum). We developed an automated segmentation pipeline based on an attention-enhanced 3D U-Net to extract all mitochondria from volumetric EM data.

**Results:**

Our quantitative analyses revealed pronounced regional and subcellular heterogeneity. In the PFC, the mitochondrial volume fraction was higher in neurites (7.2%) than in somata (2.9%; 7.1% when nucleus was excluded). Mean individual mitochondrial volume was 0.11 μm³ for neuritic and 0.33 μm³ for somatic mitochondria in the PFC, with similar results observed in the HPC (0.13 μm³ in neurites, 0.31 μm³ in somata). In both regions, the vast majority of mitochondria (~91%) assumed an oval or rod shape, with few displaying branched or donut-shaped structures (~1%). Notably, elongated linear mitochondria (~8%) were mostly confined to neurites, and approximately 90% of these comprised up to 120 nanotunnels—thin segments (<220 nm) connecting enlarged, oval-shaped structures (>350 nm) in tandem.

**Conclusion:**

These data provide a detailed quantitative characterization of mitochondrial network architecture in the adult rat cortex and hippocampus, revealing significant regional and subcellular differences in mitochondrial morphology and distribution.

## 1 Introduction

The prefrontal cortex (PFC) is a specialized brain structure that participates in a variety of higher cognitive functions such as working memory (Funahashi, 2017), thought, and emotion regulation (Woo et al., 2021). The hippocampus (HPC) is thought to participate in important cognitive functions, such as encoding episodic memories (Hartley et al., 2014). However, a common property of both hippocampus and prefrontal cortex is their functions upon memory.

Mitochondria have been reported as the powerhouse of cells (Wai and Langer, 2016). Energy is mainly derived from mitochondria at the cellular level (Picard et al., 2018). Mitochondria are involved in ATP synthesis through the tricarboxylic acid cycle and oxidative phosphorylation (van der Bliek et al., 2017). Mitochondria supply ATP to support neuronal activities, such as the generation of action potentials, and neurotransmitter release (Lin and Sheng, 2015). Mitochondrial distribution and motility are important to synapse formation (Todorova and Blokland, 2017). Mitochondria are essential to the regulation of calcium and redox (Mattson et al., 2008). Besides, mitochondria can determine axon branching and development (Courchet et al., 2013). Given these diverse roles, mitochondria may differ between principal cell types in the hippocampus and prefrontal cortex.

The size of mitochondria varies from 0.5 to 10 μm depending on host cell types and specific intracellular localization (Trushina, 2016). Different mitochondrial structures have different functional implications (Glancy et al., 2020). Depending on the cell type and location, mitochondria have different morphologies (Lin et al., 2019). “Donut-like” mitochondrial structures may be associated with oxidative stress (Ahmad et al., 2013). According to the morphology of mitochondria, mitochondria can be divided into elongated, branching, nanotunnels, donut-shaped, and oval (Glancy et al., 2020). In pyramidal neurons, dendritic mitochondria are long and tubular whereas axonal mitochondria are uniformly short (Smith et al., 2016). The mitochondrial morphological complexity of dendritic mitochondria is more complex than axonal mitochondria and somatic mitochondria based on mitochondrial complexity index in the hippocampus (Faitg et al., 2021). However, we lack a quantitative characterization of the differences in both mitochondrial size and morphology between different brain areas such as the hippocampus and prefrontal cortex.

However, both the functions of mitochondria and their abundance and distribution in neuronal compartments, have not been systematically explored. This analysis is a challenging task that requires the generation of large-volume 3D reconstructions, fortunately, recent advances in electron microscopy have greatly facilitated this task. Here, we used a powerful method, ATUM-SEM (automated tape-collecting ultramicrotome scanning electron microscopy), to map the mitochondria nanotunnels. This analysis represents an important foundation to serve for understanding the mitochondria dynamics.

## 2 Results

### 2.1 Mitochondria segmentation

**Figure 1:**
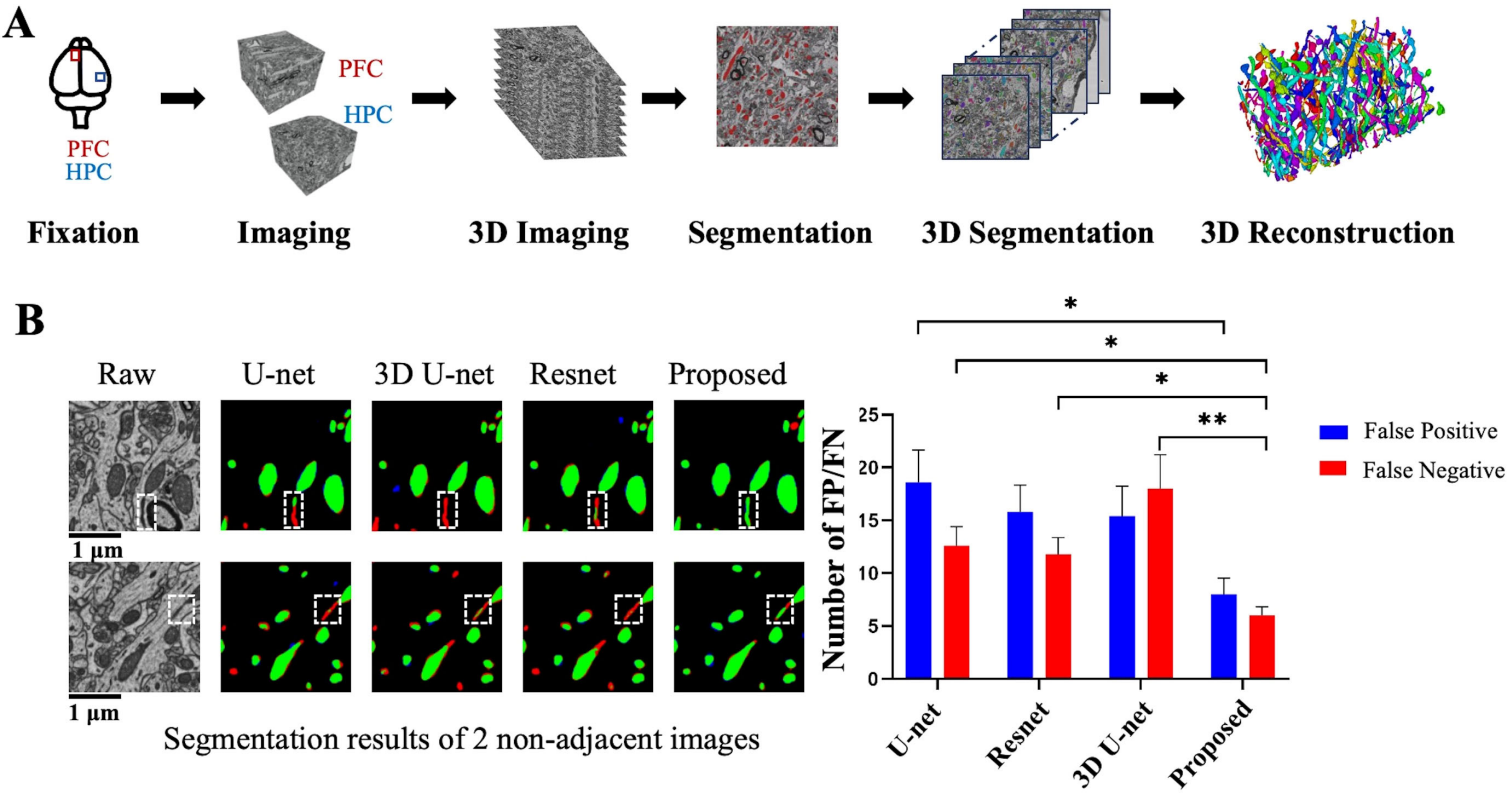
Study workflow for 3D reconstructions of mitochondria of the rat hippocampal and prefrontal cortex from EM images, and evaluation of the mitochondrial analysis method. **(A)** Pipeline of mitochondria reconstruction involving sample fixation, sample sectioning, ATUM-SEM imaging and image alignment, image segmentation and 3D connection, and 3D visualization. **(B)** Comparison of mitochondrial nanotube segmentation performance between different methods (U-net, 3D U-net, Resnet, and the proposed method) on two sections from the hippocampal dataset. Data are presented as mean ± SEM. *P < 0.05, **P < 0.01, unpaired T-test.

We acquired EM datasets from a 6-month-old rat hippocampus (Cornu Ammonis 1 stratum radiatum region) and the prelimbic cortex of the medial prefrontal cortex (**Figure 1A**) using ATUM. Images were collected at 5×5×50- or 3×3×50- nm voxel size sampling in the x, y, and z dimensions. After stitching and aligning the images, we cropped 4 sub-volumes, to make neurites(hereafter referred to collectively as neurites, which include both axons and dendrites) and somata (n≥3) be contained and avoid large blood vessels. We reconstructed hippocampal and prefrontal cortical mitochondria in the datasets (**Figure 1A**). These mitochondria constituted 7.2% and 6.3% of the total volume of neurites. To reconstruct nanotunnels, which constitute the longest wiring in the mitochondria, we applied an effective and automated pipeline based on deep learning to realize mitochondria segmentation in different EM images. We proposed a segmental method for mitochondria. The network could get information on mitochondria in serial section layers. We added a model to this network based on Resnet. In this model, the weight coefficient of a tiny part was increased (the diameter of nanotunnels was less than 220 nm). We compared the mitochondrial segmentation method proposed with other existing mitochondrial segmentation methods:U-net, 3D U-net, Resnet (Ronneberger et al., 2015; Çiçek et al., 2016; He et al., 2016).U-net is a convolutional neural network with a U-shaped architecture, featuring encoder-decoder paths. U-net is widely used for 2D image segmentation tasks, such as biomedical image analysis. 3D U-net is an extension of U-net, specifically designed for 3D data. It is mainly used for segmenting volumetric medical images. Resnet is a deep neural network with residual connections that allow training of very deep models. Resnet allows the training of very deep neural networks by introducing residual connections.

To explicitly visualize the differences between our results and the results of other methods (U-net, 3D U-net, Resnet), we displayed 2 surface-to-surface comparison examples for our datasets in **Figures 1B**, where green pixels, red pixels, blue pixels, and black pixels denote true positive (TP), false negative (FN), false positive (FP) and true negative (TN), respectively. We statistically analyzed the segmentation results of mitochondrial nanotubes obtained by our method and other methods (U-net, 3D U-net, Resnet). First, five non-adjacent 4096 × 4096 pixels images were extracted from the test set. The numbers of correctly segmented, missed, and falsely segmented mitochondrial nanotubes in these images were quantified, as shown in **Figure 1B**. Specifically, in the hippocampus dataset, which contains 207 mitochondria, our method detects 203 TPs and 5 FPs, and the missed (FN) number is 4. We further compared the segmentation results of the four different methods (U-net, 3D U-net, Resnet, and the proposed method) using the Jaccard index and Dice coefficient, as shown in **Supplementary Table 1**. The use of Jaccard index and Dice coefficient provides a more standardized basis for comparison with other studies.

Particularly, our method yields promising results in the segmentation of mitochondrial nanotunnels. As shown in the red box, our method can accurately sketch the contours of mitochondrial nanotunnels, significantly better than U-net, 3D U-net, Resnet (**Figure 1B**). It is demonstrated that our proposed method is conducive to computing and analyzing mitochondria biological statistics such as number, shape, and size.

### 2.2 Distinct mitochondrial network morphology in neuronal somata of HPC and PFC

**Figure 2:**
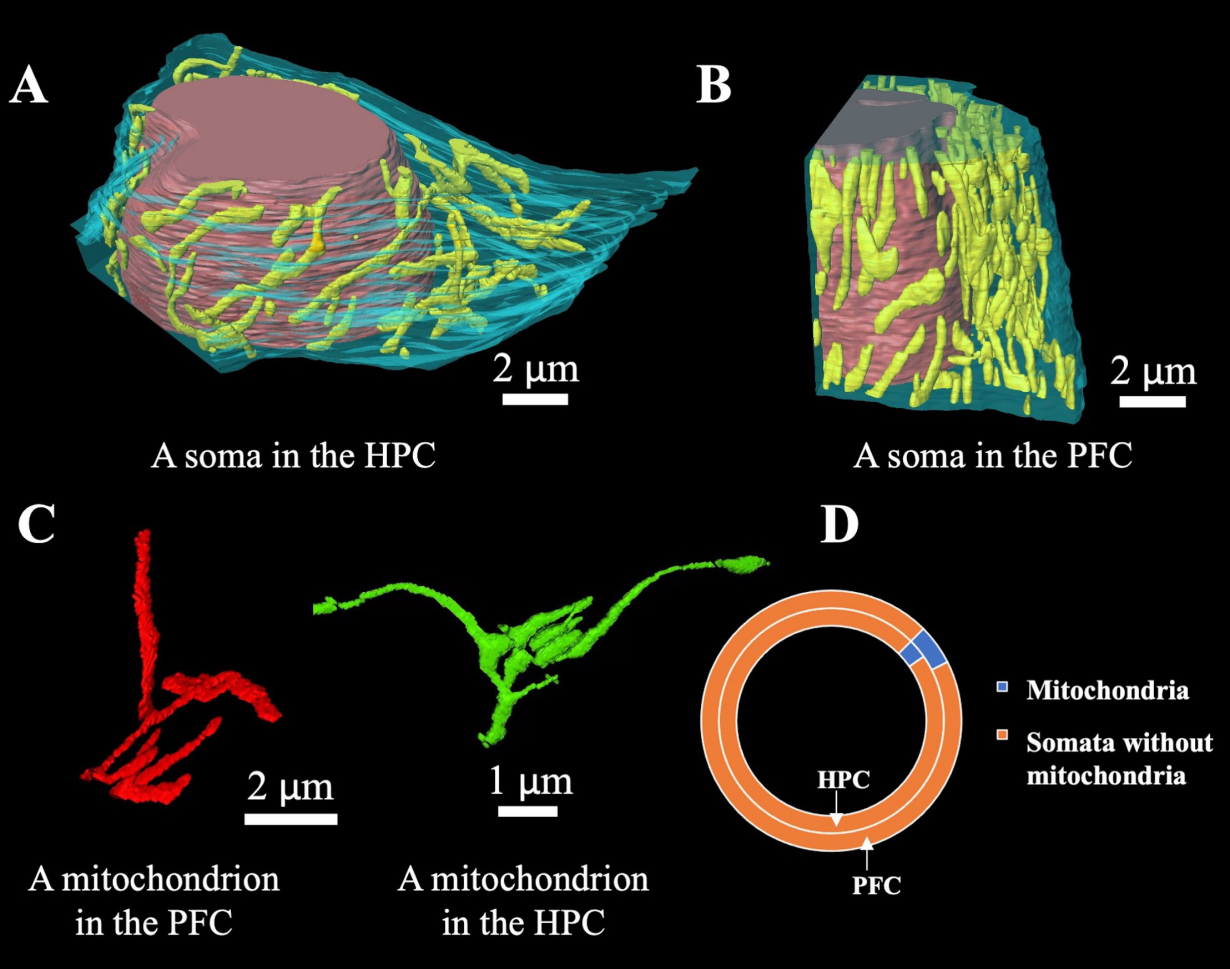
3D reconstruction of the mitochondrial network in the somata of hippocampal and prefrontal cortical neurons. **(A)** Reconstruction of mitochondria and nucleus in a soma in hippocampal neurons. The color of the mitochondria is yellow, the color of the nucleus is red, and the color of the cytoplasm is light blue. **(B)** Reconstruction of mitochondria and nucleus in a soma in prefrontal cortical neurons. The color of the mitochondria is yellow, the color of the nucleus is red, and the color of the cytoplasm is light blue. **(C)** Mitochondria in complex structures of the hippocampus. **(D)** The volume fraction of mitochondria in the hippocampus and prefrontal cortex. Length measurements were derived from 3D skeletonization, which eliminates any projection artifact and provides true 3D path lengths.

To reconstruct the mitochondrial network in the somata of hippocampal and prefrontal cortical neurons, we first selected two volumetric datasets each consisting of 100 serial EM images at a voxel size of 5×5×50 nm^3^ or 3×3×50 nm^3^ and containing multiple cell bodies (n=5 cells for the hippocampus dataset, and 3 cells for the prefrontal cortex dataset). Then, we applied the proposed method of mitochondrial segmentation to trace unbiasedly all mitochondria in their entirety. **Figure 2** shows a 3D reconstruction of a soma from the HPC (**Figure 2A**) and PFC dataset (**Figure 2B**).

Mitochondria exhibit different shapes and sizes and disperse more or less randomly and evenly in the cytoplasm surrounding the prominent nucleus. By volume, hippocampal mitochondria account for 2.9% of somata or 5.5% of the cytoplasm excluding the nucleus. In the reconstruction of the prefrontal cortex dataset, the volume density of somatic mitochondria appears to be higher, reaching 5.3% or 7.1% exclusive of the nucleus (**Figure 2D**). In particular, some mitochondria tend to form branched and tortuous network structures, with skeleton lengths reaching 38.5 μm (**Figure 2C**, red) or even exceeding 49.6 μm (**Figure 2C**, green), likely driven by dynamic fusion and fission inherent to mobile mitochondria. Quantitatively, about 16.6% mitochondrial volume was found in the complex structures, with the rest in the ovoid or tubular linear structures of 0.3-11.0 μm in length. The mitochondria in the somata of neurons in the hippocampus and prefrontal cortex are of the branching and elongated types.

### 2.3 Comparison of mitochondrial 3D structure in HPC and PFC neurites

**Figure 3:**
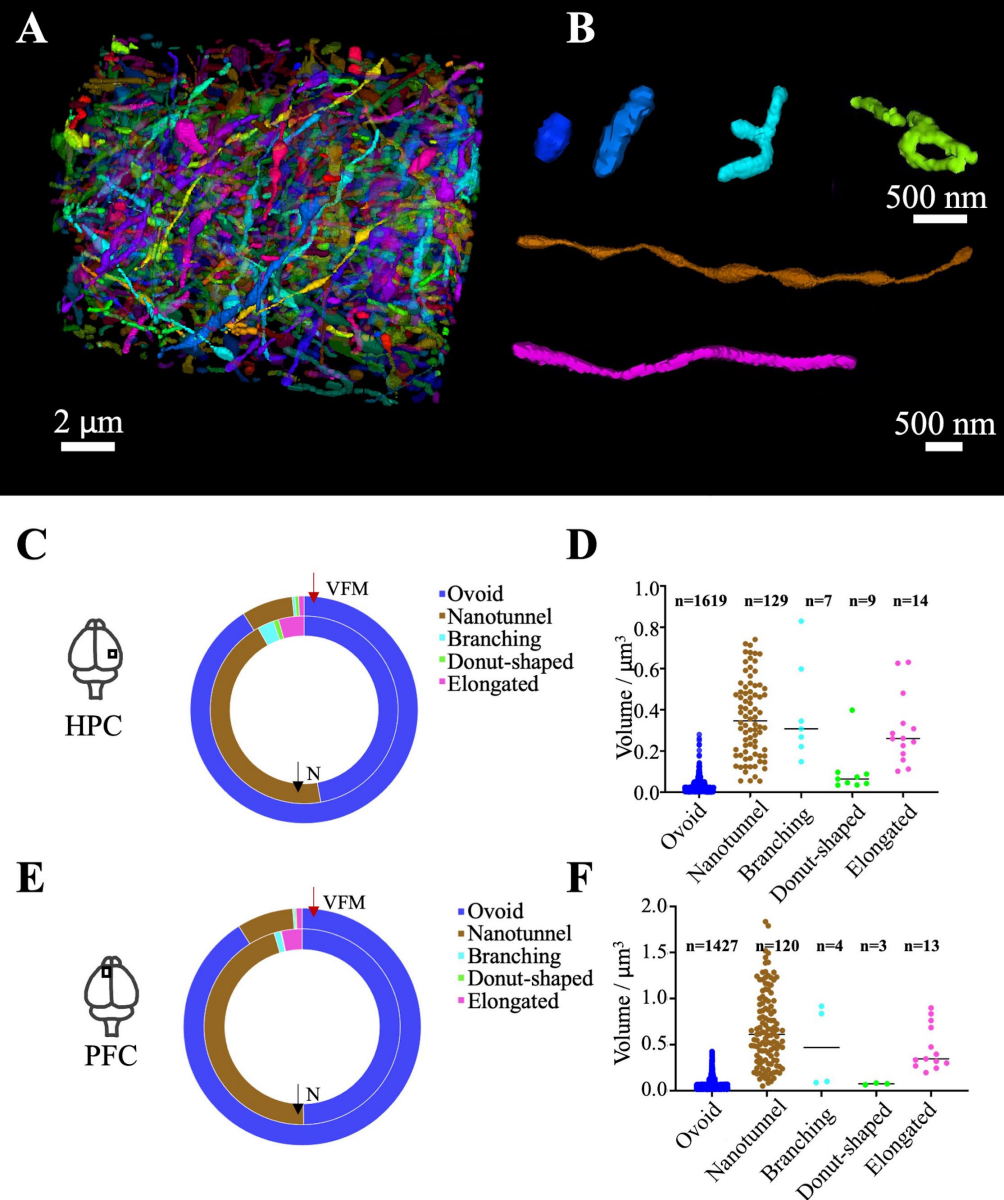
3D reconstruction of mitochondria in the neurites in the hippocampus and prefrontal cortex. **(A)** Reconstruction of all mitochondria in the neurites in the hippocampus. **(B)** Mitochondria classification. Ovoid or rod (Blue); Branching (Cyan); Donut-shaped (Green); Elongated (Pink), nanotunnels (Brown); **(C, E)** The volume fractional mass (VFM, inner ring) and number (N, outer ring) of mitochondria in hippocampus (HPC) and prefrontal cortex (PFC); **(D, F)** Distribution of mitochondrial volume in the hippocampus (HPC) and the prefrontal cortex (PFC).

In the stratum radiatum (SR) region of the CA1(Cornu Ammonis 1) region and the prefrontal cortex, we chose volumetric datasets of 20×20×15.8 μm (<ξ>3) and 18×18×15.5 μm (<ξ>3), respectively, both of which were fully occupied by neurites, devoid of neuronal somata. 3D reconstructs of the neuritic mitochondria were obtained by applying our segmentation of improved attention model (**Figures 3A, B**; **Supplementary Figure S1**). A prominent feature of neuritic mitochondria is that they distribute along the length of neurites in single-file format with seldom overlaps. In contrast to somatic counterparts, neuritic mitochondria are predominantly of linear structure, and their length exhibits a broad 0.2-14.6 μm shaped distribution with a mode at elongated and nanotunnel shape and mean value at 2.3 μm (prefrontal cortex) and 3.3 μm (hippocampus). We observed mitochondria of branching of donut-shaped morphology in relatively thick neurites (**Figure 3B**). For elongated linear mitochondria, most displayed a beaded appearance with 1–3 NT. We calculated the number (**Figures 3C, E**, inner ring), volume fraction (**Figures 3C, E**, outer ring) and distribution of volume (**Figures 3D, F**) of all kinds of mitochondria. Uniformly oval or rod mitochondria accounted for the largest proportion in both the hippocampus and prefrontal cortex. The volume of oval or rod mitochondria is small, while the volume of nanotunnels and elongated mitochondria is larger.

The mitochondria volume fraction was 7.2%, similar to that in the somatic cytoplasm (excluding the nucleus). The relative contribution of the mitochondrial mass of the ovoid, elongated linear, branching, and donut-shaped categories contribute by 91.1%, 8.1%, and 0.9%, respectively.

**Figure 4:**
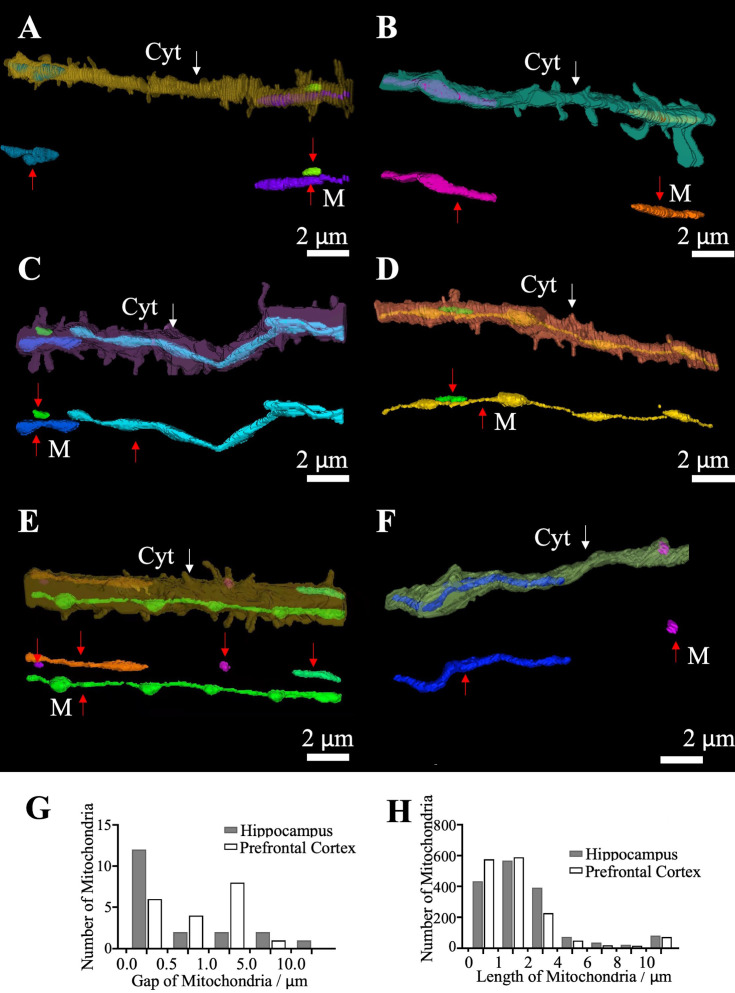
Gaps between mitochondria. **(A, B)** The gap between mitochondria in the spiny dendrite of the hippocampus **(A)** and the prefrontal cortex **(B)**. **(C, D)** Overlap between mitochondria in the spiny dendrite of the hippocampus **(C)** and the prefrontal cortex **(D)**. **(E)** A special gap of mitochondria in a dendrite in the prefrontal cortex. **(F)** Gaps between mitochondria in the smooth dendrite in prefrontal cortex. **(G)** Gap distribution. **(H)** All-length distribution of mitochondria. In the 3D reconstructions shown in **(A–F)**, mitochondria are rendered in opaque colors, while the surrounding cytoplasm is shown as semi-transparent colors surface. Red arrows indicate mitochondria and white arrows indicate cytoplasm. “M” for mitochondria and “Cyt” for cytoplasm.

The tiling of mitochondria along the shaft of neurite was not seamless: there were small gaps (spacing or distance between adjacent mitochondrial segments within a neurite) ranging from 0 to 13.0 μm between adjacent mitochondria (**Figures 4A–F**). We randomly measured the mitochondrial gaps in 20 dendrites from the hippocampus and prefrontal cortex and found that the mitochondrial gaps in the hippocampus can reach up to 13.0 µm, while those in the prefrontal cortex can extend up to 5 µm (**Figure 4G**).

As a result, up to 40%-100%, the entire length of neurite is covered by mitochondria largely centered in the shaft of neurites, and the longest distance of the plasma membrane to the nearest mitochondrial membrane is no more than 1.5 μm in the present dataset, allowing for diffusive messengers and metabolites to communicate speedily between the mitochondria and the surface membrane including associated structures such as postsynaptic spines. We didn’t see any mitochondria in spines.

The spacing between mitochondria may be optimized to support different types of synaptic inputs. In a study on the ferret visual cortex, it was found that mitochondria in dendrites are distributed in a manner that supports functional diversity rather than just high synaptic activity (Thomas et al., 2023).

To address this, we performed a 3D skeletonization of all reconstructed mitochondria in both the PFC and HPC datasets. We extracted the centerline (path length) of each mitochondrion, regardless of its orientation. This approach ensures that our length measurements are independent of projection artifacts and truly reflect the 3D morphology. The results of this quantitative analysis are now presented in new **Figure 4F**.

HPC (Hippocampus): Mitochondria exhibited a mean skeleton length of 3.3 µm, with 84.5% of mitochondria having a skeleton length of less than 4 µm. A minority of mitochondria exceeded 10 µm in size (0.05%). PFC (Prefrontal Cortex): Mitochondria showed a mean skeleton length of 2.4 µm, with 89.7% of mitochondria having a skeleton length of less than 4 µm. Only 0.04% of mitochondria exceeded 10 µm in length.

### 2.4 Quantitative analysis of mitochondrial nanotunnels

Mitochondrial nanotunnels were first described in rat cardiac myocytes and named on the basis of their structure as long, thin, double-membrane extensions. In live cells under optical microscopy, fluorescence-stained mitochondrial nanotunnels are dynamic structures that are actively protruded from single mitochondria and extend to others separated at several micron distances. Functionally, nanotunnels conferred an effective means for inter-mitochondrial communication among the entire cohort of mitochondria immobilized amidst lattice-like myofibrils (Huang et al., 2013).

In brain tissues, mitochondrial nanotunnels are usually called mitochondria-on-a-string, and the thin segments are intermingled with enlarged parts. It was found in hippocampus from human and mouse models of Alzheimer’s disease (Zhang et al., 2016). Similar MOAS (mitochondria-on-a-string) mitochondrial phenotypes were described in the Dorsolateral prefrontal cortex of rhesus macaques, and mitochondrial morphology changes are age-related (Morozov et al., 2017). One reason for the formation of MOAS is that calcium overloaded, then leads to the dysfunction of mitochondria, resulting MOAS (Woo et al., 2021). Another reason about MOAS was that it may be associated with energetic stress, and hypoxic conditions could promote MOAS formation (Trushina, 2016). Other reasons for MOAS were that under stress conditions, fission arrest may promote the mitochondrial residual functioning (Pérez et al., 2017).

The single-file organization of neuritic mitochondria might suggest that mitochondrial nanotunnels would be of little motility. We supposed that the mitochondrial nanotunnels reconstructed in neurites might also serve for dynamic communication among motion-restricted mitochondrial cohorts. In this regard, we observed no somatic mitochondrial nanotunnels in the hippocampus and only a few (~0.7%) in the cell bodies of the prefrontal cortex.

**Figure 5:**
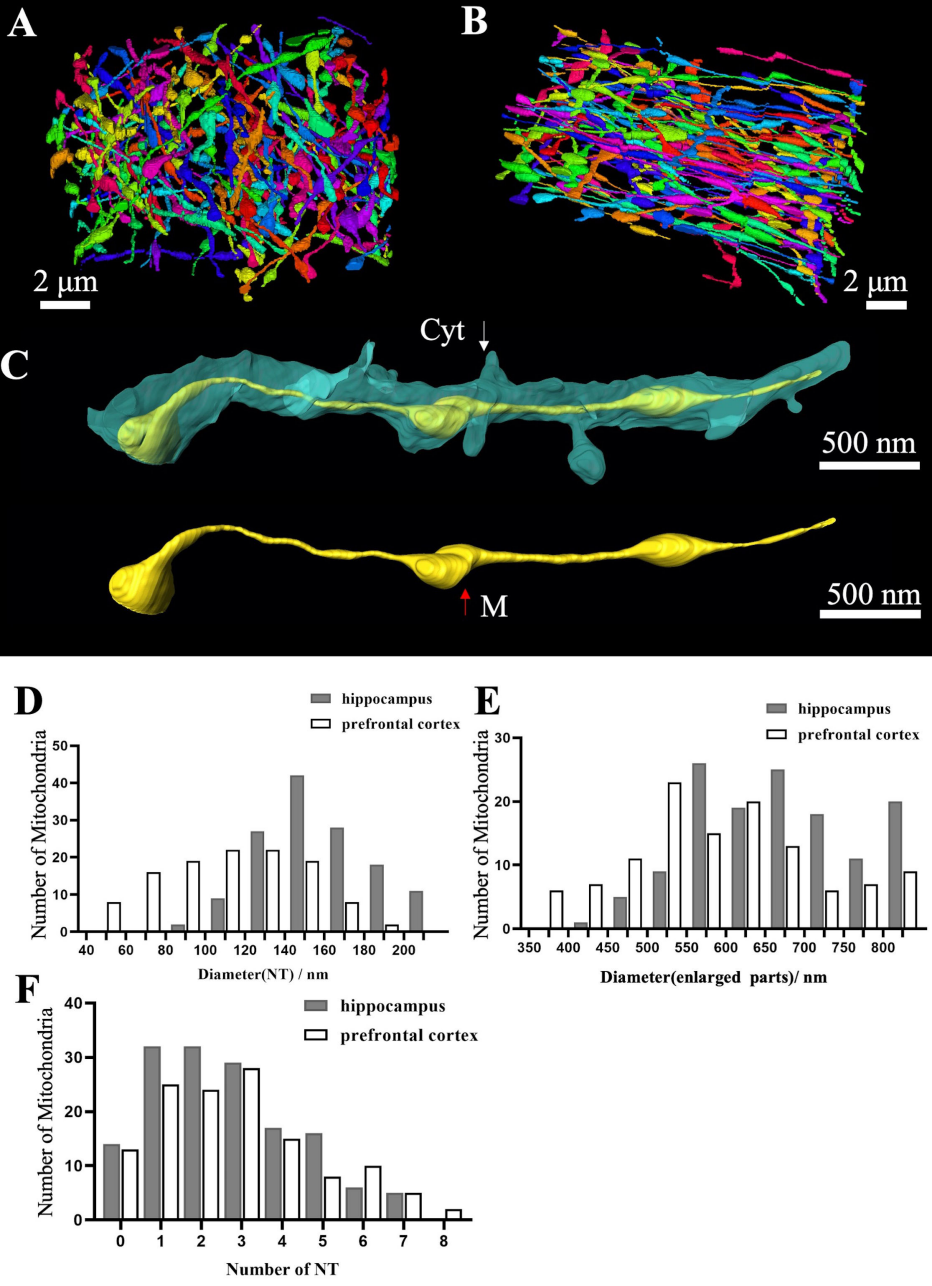
Quantitative statistics of MOAS in the hippocampus and prefrontal cortex **(A)** Mitochondria nanotunnels in the hippocampus. **(B)** Mitochondria nanotunnels in the prefrontal cortex. **(C)** Nanotunnels in a spiny dendrite. **(D, E)** Diameter of nanotunnel parts and enlarged parts. **(F)** Number of nanotunnel parts. Red arrows indicate mitochondria and white arrows indicate cytoplasm.

All elongated mitochondria with nanotunnels within neurites were traced and analyzed. The 3D reconstruction revealed that these mitochondria in neurites exhibited an elongated linear structure with a beaded appearance analogous to the mitochondrial nanotunnels reported previously (**Figures 5A–C**; **Supplementary Figure S1**). The skeleton length of these mitochondria in the hippocampus was 11.5-37.5μm, and that of prefrontal cortical mitochondria was 12.7-30.5μm (n = 129 mitochondria in the hippocampus, and n = 120 mitochondria in the prefrontal cortex; **Figures 5A, B**). The contribution of the mitochondrial mass of the nanotunnel categories contributes by 7.2% in the hippocampus, similar to 7.6% in the prefrontal cortex. The average volume density of prefrontal cortical nanotunnels was 45.6%, similar results were observed for hippocampal mitochondria (44.6%).

The cross-section diameters of the thin parts were < 200 nm, while that of the beaded segments were >350 nm (**Figures 5D, E**). The majority of mitochondria were elongated along dendrite shafts, revealing uniform diameters of 350–800 nm throughout the entire length of the mitochondria (**Figures 5D, E**). Nanotunnel (NT) and the beaded mitochondrial segments show a mosaic distribution, such as three beaded segments and three nanotunnels as shown in **Figure 5C**. For these elongated linear mitochondria, 65% displayed a beaded appearance with 1–3 NT and about 1.7% of them comprised up to 8 thin tubules in the prefrontal cortex (**Figure 5F**).

## 3 Discussion

Determining the abundance and extension of nanotunnels by an ultrastructural analysis is the key to understanding mitochondria dynamics. Here, we used ATUM to analyze the 3D structure of the mitochondria and mitochondria nanotunnels, and the proportion of them in different brain tissues, such as the hippocampus and prefrontal cortex. First of all, we reconstructed all mitochondria in the hippocampus and prefrontal cortex. Secondly, the 3D model facilitated a systematic analysis of the distribution and abundance of mitochondria. In addition, the pixel resolution of ATUM-SEM (3×3×50 nm (Hartley et al., 2014) or 5×5×50 nm (Hartley et al., 2014)) was sufficient for recognizing the membranes of the mitochondria. We developed an automated segmentation pipeline based on an attention-enhanced 3D U-Net to extract all mitochondria from volumetric EM data. The primary advantage of our proposed framework is its enhanced sensitivity to fine, branching structures (nanotunnels < 220 nm) compared to conventional 3D U-Net, which tends to smooth out such connections. The model performs optimally on datasets with consistent contrast and minimal staining variations. While designed for mitochondria, this architecture may be transferable to other tasks requiring segmentation of thin tubular structures (e.g., endoplasmic reticulum). The main drawback of the ATUM-SEM technique is the relatively low z-axis resolution (~50 nm), which leads to the inability to visualize some ultra-structures. However, for biological structures over a hundred nanometers in size, the impact can be ignored. While our cross-validated results within a single dataset demonstrate the robustness of our method, we acknowledge that the ultimate test of generalizability requires validation on independent datasets acquired under different conditions or from different laboratories. Future work should focus on applying our attention-based framework to publicly available EM volumes to further establish its broad applicability.

A potential limitation of this study is the use of a single age group (6-month-old rats). Mitochondrial structure and function are known to be dynamic across the lifespan, with significant changes reported during development and aging (Picard et al., 2014). Therefore, our findings may not generalize to younger or older animals. Future studies incorporating multiple age cohorts (e.g., postnatal, middle-aged, and aged) will be necessary to determine whether the regional differences in mitochondrial network morphology observed here are stable or undergo age-dependent modulation.

In the hippocampus and prefrontal cortex, mitochondria are densely around the intracellular space and exhibit a broad variety of mitochondrial shapes. By volume, hippocampal mitochondria account for 2.9% of somata or 5.5% of the cytoplasm excluding the nucleus. The volume density of somatic mitochondria appears to be higher, reaching 5.3% or 7.1% exclusive of the nucleus, and about 16.6% of them are in complex structures. Surprisingly, mitochondria in somata tend to form branched and tortuous network structures whose dimensions may extend greater than 49.6 μm. Nanotunnels in somata were only found in the prefrontal cortex (2.0%). Neuronal somata are the primary sites of protein synthesis (via rough ER and ribosomes) and transcriptional activity. These processes require a substantial and sustained supply of ATP, as well as calcium buffering capacity. Therefore, the higher density of mitochondria in the soma, including complex, branched networks, likely reflects the need to support these housekeeping functions. The ‘tortuous’ network morphology we observed may facilitate efficient energy distribution and calcium sequestration throughout the voluminous cytoplasm surrounding the nucleus (Vincent et al., 2017).It has been demonstrated that neuronal somata perform higher levels of aerobic glycolysis and lower levels of oxidative phosphorylation than terminals, both during basal and activated states (Wei et al., 2023).

In contrast, axons are specialized for action potential propagation and neurotransmitter release at synaptic terminals. These processes involve rapid, localized changes in energy demand and calcium influx. The elongated, often rod-shaped mitochondria we observed in neurites, with their smaller individual volumes but higher length-to-width ratios, may be optimized for transport along microtubules (Saxton and Hollenbeck, 2012).

Mitochondria exhibited a broad variety of mitochondrial shapes, we classified them as elongated, branching, nanotunnels, donut-shaped, uniformly oval or rod mitochondria (Glancy et al., 2020). At the same time, we calculated the number and volume fraction of mitochondria in the hippocampus and the prefrontal cortex. In the hippocampus, uniformly oval or rod mitochondria accounted for the largest proportion 91.1%. A single nanotunnel mitochondria almost had the largest volume and accounted for ~45% in total by volume. Similar results were observed for the prefrontal cortical mitochondrial network. The number difference of NT in the hippocampus was nearly identical to the difference observed in the prefrontal cortex. Mitochondrial nanotunnels in the prefrontal cortex were smaller with a narrow size distribution, whereas in the hippocampus are larger by testing the diameter and the length. We also found that a lot of neuritic mitochondria distribute along the length of neurites in a single-file format. Mitochondria in neurites were different from that in somata, they were predominantly of linear structure. Mitochondria exchange Ca^2+^ and reactive oxygen species with each other as well as with the endoplasmic reticulum (Booth et al., 2016). In addition, it has been shown that ER–mitochondria contacts coordinate mtDNA replication with mitochondrial division in yeast and human cells (Murley et al., 2013; Lewis et al., 2016).

In recent years, mitochondrial nanotunnels have been studied extensively in skeletal and cardiac muscles. The importance of mitochondrial nanotunnels has attracted great attention over the years. Usually, nanotunnels are studied with fluorescence images. We achieved mitochondrial images with electron microscopy, reconstructed mitochondrial nanotunnels morphology, and analyzed the difference of nanotunnels in the hippocampus and prefrontal cortex. Nanotunnels are double-membrane protrusions that involve both the inner and outer mitochondrial membranes. Some of them can be observed to be blunt-ended, and some of them are nanotunnels. Nanotunnels could be found in the hippocampus and cortex and have been observed in rat cardiomyocytes, human skeletal muscle, rat skeletal muscle, and African green monkey kidney cells (Vincent et al., 2017). Mitochondrial nanotunnels form and elongate in a kinesin (KIF5B)- and microtubule-dependent manner within seconds (Wang et al., 2015). In the heart, Huang, et al. have reported that inter-mitochondrial communication occurred between pairs of well-separated mitochondria in a saltatory fashion, bypassing their intermediate neighbors. And a mitochondrion could communicate with a remote mitochondrion about 8 μm away (Huang et al., 2013). We did not perform relevant experiments to confirm the dynamic process of nanotunnels in these regions. At the distal end, they taper into a rounded shape but, despite considerable effort in this regard, we were not able to observe a direct continuity between the far end of a nanotunnels and a receiving mitochondrion. Because of the lack of mouse brain samples from disease models, we will further compare the abundance of nanotunnels between healthy and disease neurons in our subsequent studies.

An intriguing question raised by our findings is whether the regional differences in nanotunnel frequency are influenced by the local cellular environment. Given that nanotunnel formation is calcium-dependent, the endoplasmic reticulum (ER)—as the primary intracellular calcium store—is a likely modulator. Previous studies have demonstrated regional heterogeneity in ER morphology and distribution across brain areas (Spacek and Harris, 1997). It is tempting to speculate that the higher nanotunnel density in HPC neurites may be supported by a more elaborate ER network or more frequent mitochondria-ER contacts in this region, facilitating the calcium transients required for nanotunnel formation. Future correlative studies combining mitochondrial and ER segmentation in the same datasets will be necessary to test this hypothesis directly.

In summary, our results expand the knowledge about the 3D structure and abundance of the various organelles in different brain tissues of rat. They also enable us to determine the nanotunnels at a nanometer resolution. However, our results only present qualitative and quantitative information from a structural perspective based on static imaging. Therefore, our findings still require further EM and dynamic imaging to verify their uniqueness, including the fission and fusion of the outer and inner mitochondrial membrane. Future studies combining high-resolution 3D ultrastructure with functional imaging (e.g., calcium or ATP sensors) in identified neuronal compartments will be necessary to directly test the hypothesis that the morphological differences we observe translate into distinct functional capabilities.

## 4 Methods

### 4.1 Animals

All rats were on a 12-h light/dark cycle with standard rat chow and water ad libitum unless otherwise noted. We used IACUC (Institutional Animal Care and Use Committee) at Tsinghua University (15-LB 5) in our research, and all the experiments were carried out according to the guidelines of AAALAC (Association for Assessment and Accreditation of Laboratory Animal Care International).

### 4.2 Sample fixation, sample sectioning, and wafer fabrication

The PFC samples were immersed in 4% (w/v) PFA and 2.5% (Sigma, G5886) GA. Then, the samples were fixed in 2% OsO4 (Ted Pella, 18451) in phosphate buffer (0.1M, pH 7.4) for 90 mins at room temperature. After being washed by 0.1 M PB buffer, the brain samples were treated with filtered thiocarbohydrazide (TCH, Sigma, 223220) for 45 min at 40 °C. After that, the samples were post-fixed by 2% OsO4 for 90 min followed by incubated with 1% uranyl acetate aqueous solution at 4 °C overnight. Then, the sample was dehydrated with a gradient ethanol series (50%, 70%, 80%, 90%, and 100% ethanol, 10 min for each) and pure acetone. Finally, the samples were ultimately embedded with Epon 812 resin (SPI, 02660-AB).

### 4.3 ATUM-SEM imaging

Serial sections of the hippocampus and prefrontal cortex samples were continuously cut and collected by a diamond knife (Diatome, MC16425) using the automated tape-collecting ultramicrotome (Hayworth et al., 2014) system (ATUM-SEM). The cutting speed is 0.8-1.0 mm/s. The collection Kapton polyimide tape is 8 mm wide and 100 μm thick.

### 4.4 Image alignment

The serial sections of the PFC sample were continuously cut with a diamond knife (Diatome, MC16425) using the ATUM system. Then we acquired the serial section images through a scanning electron microscope (SEM). Due to the discontinuity and nonlinear deformation of serial section images introduced by slicing process, image alignment is necessary to restore the 3D structure of the biological tissue.

So, we adopted a coarse-to-fine strategy to align the serial section images. Firstly, we extract corresponding points and use affine transformations to estimate the positional relationship between adjacent sections. Then, the fine alignment (Xin et al., 2023) was performed to correct the nonlinear deformation of serial section images. Finally, the 3D EM image stack was obtained. Thus, we acquired a stack of 300 images (4000×4000 pixels), with a corresponding size of approximately 20×20×31.5 μm (Hartley et al., 2014).

### 4.5 Dataset annotation

Firstly, we manually annotated 64 pieces of pictures, 50 of them are trained by our proposed model to predict binary masks of mitochondria. We employed a 3D connection method to calculate the relationship between mitochondria in serial slices (Li et al., 2018). Then the prediction was proofread and modified by three neuroscience experts.

### 4.6 Segmentation

We performed ATUM on samples of the rat. We observed tissue samples from the hippocampus and the prefrontal cortex of rats by scanning electron microscope. Images were collected at 3×3×50 nm (Hartley et al., 2014) or 5×5×50 nm (Hartley et al., 2014) voxel size sampling in the x, y, and z dimensions. In this dataset, the mitochondria were annotated in two volumes: training volume and testing volume. The training dataset consists of a stack of 50 slices from the hippocampus datasets and prefrontal cortex datasets. The ground truths were annotated by neuroanatomists using Fiji (Schindelin et al., 2012) with the TrakEm2 (Cardona et al., 2012) plug-in. The production of such a ground truth database required a great amount of human effort. In pre-processing, we presented the pre-processing method consisting of image registration and histogram equalization. Based on 3D supervised convolutional network (Xiao et al., 2018), we came up with a new framework with an attention model combined, to extract 3D information from serial EM data effectively. Next, we applied our well-trained model to the large-scale ATUM-SEM hippocampus and prefrontal cortex datasets, which consist of 20×20×15.8 μm (Hartley et al., 2014) volume and 18×18×15.5 μm (Hartley et al., 2014) volume, respectively. Then, we employed a 3D connection method to calculate the relationship between mitochondria in serial slices (Li et al., 2018).

Our proposed model is built upon a 3D U-Net architecture as the backbone. The network consists of 4 encoding and 4 decoding layers with skip connections. We incorporated a spatial attention module after the third down-sampling layer to force the network to focus on the thin, elongated structures of mitochondrial nanotunnels. To address the class imbalance (i.e., nanotunnel voxels are far fewer than background or mitochondrial body voxels), we implemented a weighted loss function.” We assigned a higher weight coefficient (*α*) to the nanotunnel class during training. The weights were empirically set based on the volume ratio: α(tunnel)=0.6, α(mitochondria)=0.3, α(background)=0.1.

### 4.7 3D visualization and quantification

The connection maps were imported into ImageJ and Amira software (Stalling et al., 2005) for 3D visualization of the mitochondria. From the 3D reconstruction results, it can be seen that most mitochondria are intact and continuous, which shows the validity and feasibility of our proposed network and 3D connection method. In addition, some morphological measurements of the organelles were obtained through Amira software, such as 3D volume and 3D surface area.

### 4.8 Statistical analysis

All statistical analyses were performed using GraphPad Prism. Data are presented as mean ± standard error of the mean (SEM), as indicated in the figure legends. For comparisons between two groups (e.g., hippocampus vs. prefrontal cortex), we used two-tailed unpaired t-tests when data met the assumptions of normality and homogeneity of variance. The specific statistical test used for each analysis is indicated in the corresponding figure legend. Statistical significance was defined as *p < 0.05, **p < 0.01, and ***p < 0.001.

In our study, classification was performed based on 3D reconstructions of individual mitochondria, using the following criteria: Oval/rod-shaped: Length-to-width ratio < 3, no branches. Elongated: Length-to-width ratio ≥ 3, no branches. Branched: Presence of at least one branch point. Donut-shaped: Mitochondrion forms a closed ring structure. Nanotunnels: Thin (<220 nm) interconnections between two or more mitochondrial compartments.

## Data Availability

The datasets presented in this article are not readily available because Data are available from the corresponding author on reasonable request. Requests to access the datasets should be directed to luwang@ihep.ac.cn.

## Supplementary material

Supplementary Table 1 In this study, 14 ATUM-SEM images of the rat hippocampus, each with a size of 4000 × 4000 pixels, were used as the test dataset for mitochondria.

Supplementary Figure S1 Raw electron microscopy images and corresponding 3D reconstructions of mitochondria **(A)** Raw electron microscopy images. **(B)** 3D reconstructions of mitochondria corresponding to **(A)**.
