# Supplementary material for: Quantitative reconstruction of neuronal mitochondrial network in neurites and somata in rat hippocampus and prefrontal cortex
Source: Front Physiol. 2026 Apr 21;17:1735677. doi: 10.3389/fphys.2026.1735677 (PMC13139008; doi:10.3389/fphys.2026.1735677)
Supplement: Supplementary Table 1 — In this study, 14 ATUM-SEM images of the rat hippocampus, each with a size of 4000 × 4000 pixels, were used as the test dataset for mitochondria. [file SupplementaryFile1.docx]

**Supplementary Table 1**

In this study, 14 ATUM-SEM images of the rat hippocampus, each with a size of 4000 × 4000 pixels, were used as the test dataset for mitochondria. The segmentation results were evaluated using the Jaccard index and Dice coefficient, which are commonly used metrics for assessing image segmentation performance. Jaccard index: Given two sets X and Y, the Jaccard index is defined as the ratio of the size of their intersection to the size of their union. Dice coefficient (also known as F1 score): commonly used to calculate the similarity between two sets, X and Y.

$$\begin{aligned} Jaccard\left( X,Y \right)=\frac{X\cap Y}{X\cup Y}\# \end{aligned}$$

$$\begin{aligned} Dice\left( X,Y \right)=\frac{2\times Jaccard\left( X,Y \right)-1}{Jaccard\left( X,Y \right)}\# \end{aligned}$$

Evaluation metrics for mitochondrial segmentation

|  | U-net | 3D U-net | 3D ResNet | Proposed |
| --- | --- | --- | --- | --- |
| Jaccard index | 0.6537 | 0.5978 | 0.7436 | 0.7607 |
| Dice coefficient | 0.7836 | 0.6857 | 0.8529 | 0.8641 |

**Supplementary Figure**


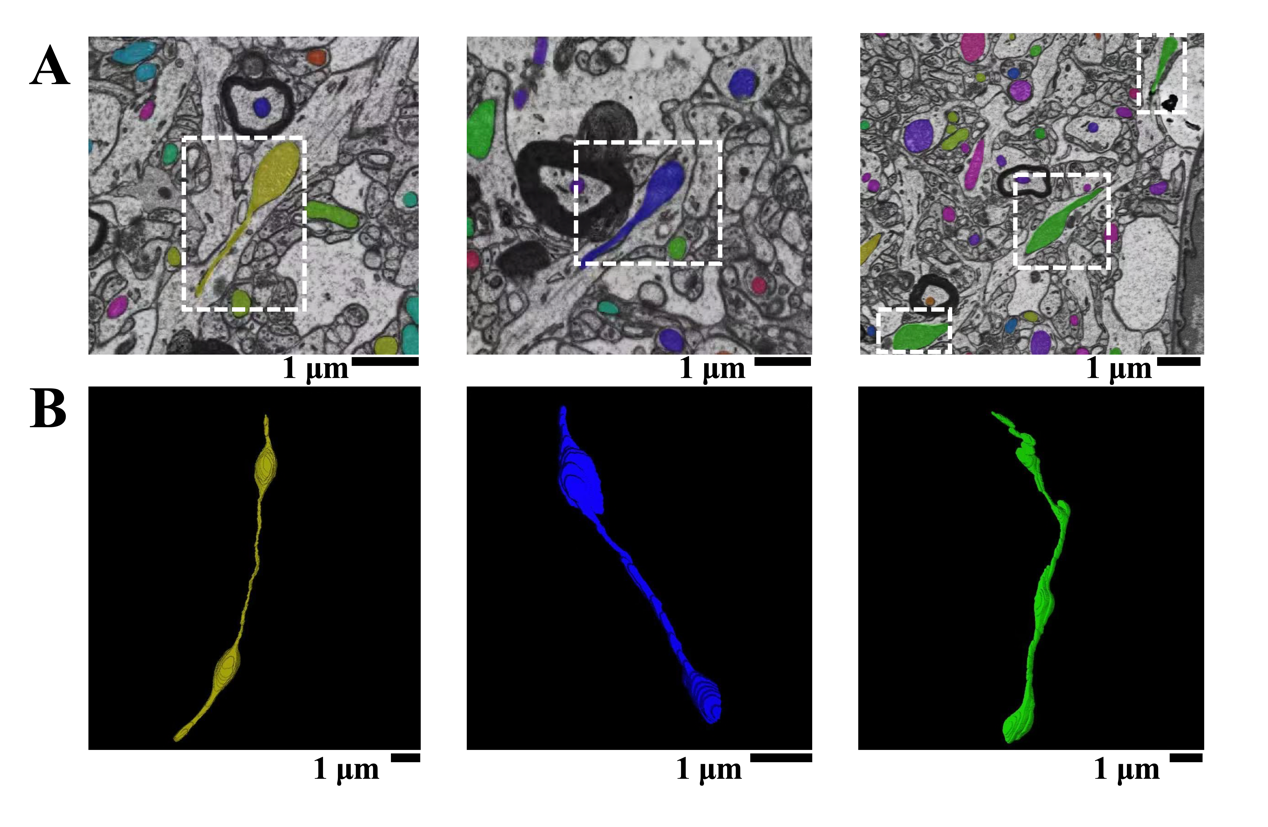


Figure S1. Raw electron microscopy images and corresponding 3D reconstructions of mitochondria

(A) Raw electron microscopy images. (B) 3D reconstructions of mitochondria corresponding to A
